# Inflammatory bowel disease is associated with an increase in the incidence of multiple sclerosis: a retrospective cohort study of 24,934 patients

**DOI:** 10.1186/s40001-024-01776-w

**Published:** 2024-03-20

**Authors:** Kaneschka Yaqubi, Karel Kostev, Isabel Klein, Sofia Schüssler, Petra May, Tom Luedde, Christoph Roderburg, Sven H. Loosen

**Affiliations:** 1 Clinic for Gastroenterology, Hepatology and Infectious Diseases, Medical Faculty, University Hospital Düsseldorf, Heinrich Heine University Düsseldorf, Moorenstraße 5, 40225 Düsseldorf, Germany; 2 Epidemiology, IQVIA, Frankfurt, Germany

**Keywords:** IBD, Ulcerative colitis, UC, Crohn’s disease, CD, MS, Neurodegenerative disease

## Abstract

**Background:**

Recent data suggest a potential pathophysiological link between inflammatory bowel disease (IBD) and multiple sclerosis (MS), two immune-mediated diseases both of which can have a significant impact on patients' quality of life. In the present manuscript, we investigate the association between IBD and MS in a German cohort of general practice patients. These results may have important implications for the screening and management of patients with IBD, as well as for further research into the pathophysiological mechanisms underlying both disorders.

**Methods:**

4,934 individuals with IBD (11,140 with Crohn’s disease (CD) and 13,794 with ulcerative colitis (UC)) as well as 24,934 propensity score matched individuals without IBD were identified from the Disease Analyzer database (IQVIA). A subsequent diagnosis of MS was analyzed as a function of IBD using Cox regression models.

**Results:**

After 10 years of follow-up, 0.9% and 0.7% of CD and UC patients but only 0.5% and 0.3% of matched non-IBD pairs were diagnosed with MS, respectively (p_CD_ = 0.002 and p_UC_ < 0.001). Both CD (HR: 2.09; 95% CI 1.28–3.39) and UC (HR: 2.35; 95% CI 1.47–3.78) were significantly associated with a subsequent MS diagnosis. Subgroup analysis revealed that the association between both CD and UC and MS was more pronounced among male patients.

**Conclusion:**

The results of our analysis suggest a notable association between IBD and a subsequent MS diagnosis. These findings warrant further pathophysiological investigation and may have clinical implications for the screening of IBD patients in the future.

## Key summary

Summarise the established knowledge on this subject

- There is a growing body of evidence suggesting a potential pathophysiological link between inflammatory bowel disease (IBD) and multiple sclerosis (MS).
- However, epidemiological data of large real-word cohorts on this potential association are scarce
- In the present manuscript, we investigate the association between IBD and MS in a large cohort of over 24.000 outpatients in Germany.

What are the significant and/or new findings of this study?

- Within a 10-year follow-up period, the incidence of MS was significantly higher among Crohn’s disease (CD, 0.9% vs 0.5%) and ulcerative colitis (UC) patients (0.7% vs 0.3%) compared to matched non-IBD patients.
- Both CD (HR: 2.09; 95% CI 1.28–3.39) and UC (HR: 2.35; 95% CI 1.47–3.78) were significantly associated with a subsequent MS diagnosis in regression analysis.
- Subgroup analysis revealed that the association between both CD and UC and MS was more pronounced among male patients.
- These findings warrant further pathophysiological investigation and may have clinical implications for the screening of IBD patients in the future.

## Introduction

Inflammatory bowel disease (IBD) and multiple sclerosis (MS) are two chronic inflammatory diseases. IBD is characterized by inflammation of the gastrointestinal tract and comprises both ulcerative colitis (UC) and Crohn’s disease (CD). The prevalence of both UC and CD has been on the rise and currently affects up to 505 per 100,000 and 322 per 100,000 individuals in Europe, respectively, without sex-specific predominance [1, 2]. MS is a complex autoimmune disease that affects the central nervous system and is characterized by demyelination, neuroinflammation and neuroaxonal degeneration. It has an estimated prevalence of approximately 0.1% and disproportionately affects women, with a female to male ratio of 3:1 [3].

Although there is evidence of an association between MS and IBD [4], the underlying reasons and mechanisms for this epidemiological association are far from understood. Shared features, such as genetic predispositions, environmental factors, and immunological dysregulation, suggest a potential biological link between these conditions [5–7]. However, a comprehensive understanding of the mechanisms underlying this association remains elusive.

Understanding the association between MS and IBD is crucial due to its potential impact on clinical management and patient outcomes. Several studies have suggested that the co-occurrence of these conditions could worsen disease outcomes, increase the risk of disability, and adversely affect quality of life [8]. Moreover, recent evidence suggests that the presence of IBD may increase the risk of developing MS [9].

In this study, we aim to investigate the association between IBD and a subsequent diagnosis of MS in a large cohort of patients with IBD in a general practice setting in Germany. Our findings may offer important implications for the management and treatment of patients with IBD and MS.

## Materials and methods

### Database

The present retrospective cohort study was based on data from the Disease Analyzer (DA) database (IQVIA), which has already been used in several previous studies focusing on IBD or MS [10–12]. It contains anonymous data on diagnoses, prescriptions, as well as basic medical and demographic patient data derived from computer systems in office-based practices in Germany [13]. The DA database covers approximately 3–5% of all office-based practices in Germany. Statistics from the German Medical Association are used as a sampling method to define a representative panel design according to specialist group, German federal state, community size category, and age of physician [13].

### Study population

**Fig. 1:**
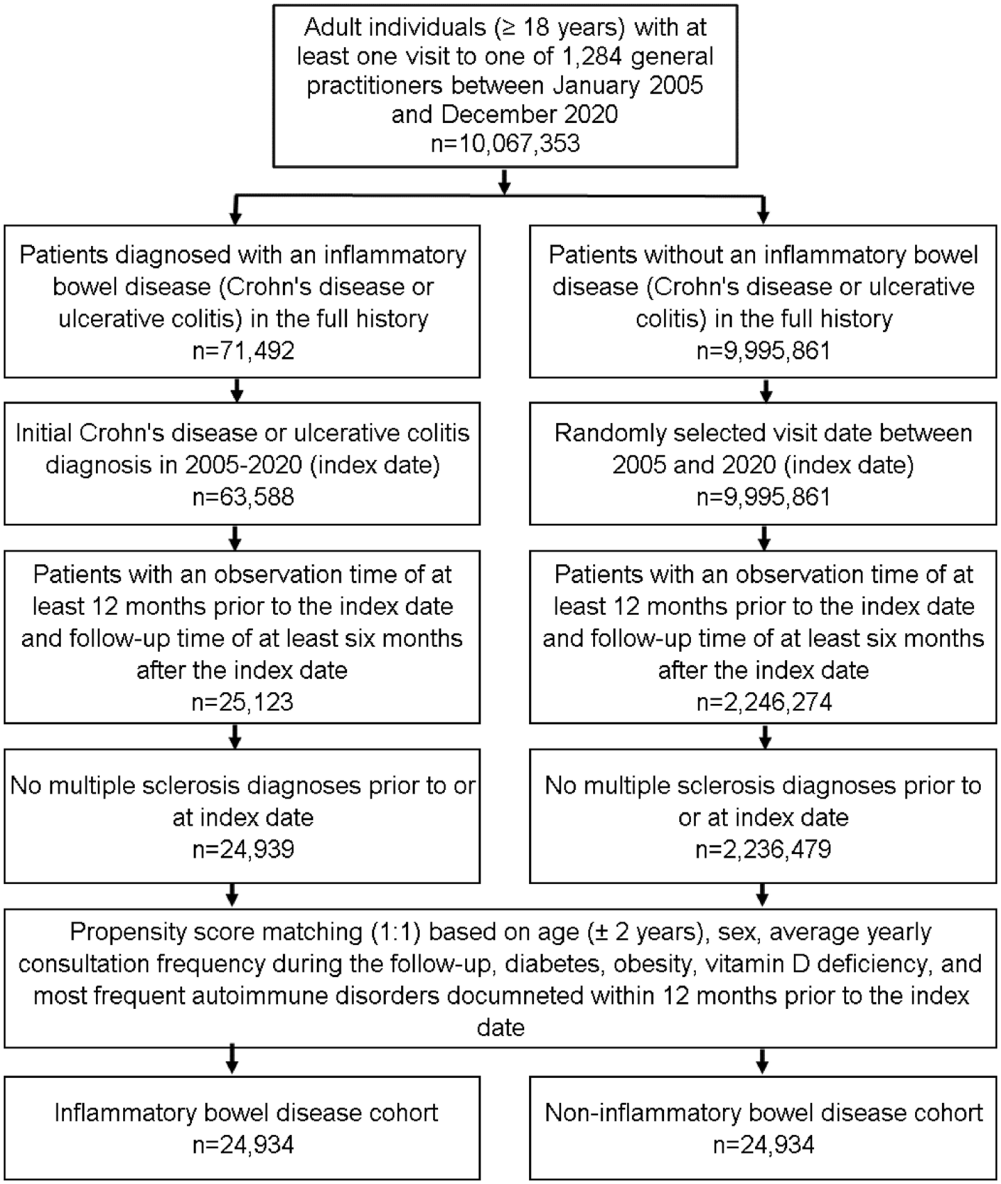
Selection of study patients

This study included patients aged ≥ 18 years with an initial IBD diagnosis including Crohn’s Disease (ICD-10: K50) and ulcerative colitis (ICD-10: K51) from 1,284 general practices in Germany between January 2005 and December 2020 (index date; Fig. 1). Further inclusion criterion was an observation time of at least 12 months prior to the index date and a follow-up time of at least six months after the index date. Patients with diagnoses of multiple sclerosis (ICD-10: G35) prior to or at index date were excluded. After applying similar inclusion criteria, individuals without IBD were matched to IBD patients using greedy nearest neighbor propensity score matching (1:1) based on age (± 2 years), sex, average yearly consultation frequency during the follow-up, diabetes, obesity, vitamin D deficiency, and most frequent autoimmune disorders (rheumatoid arthritis, psoriasis, atopic dermatitis, autoimmune thyroiditis, ankylosing spondylitis, systemic lupus erythematosus, celiac disease, thrombophilia) as group documented within 12 months prior to the index date. The matched cohorts were considered balanced when there were no statistically significant differences between variables included in matching (*p* < 0.05). For the non-IBD cohort, the index date was that of a randomly selected visit between January 2005 and December 2020 (Fig. 1). Diabetes, obesity, vitamin D deficiency and autoimmune disorders were included as these diagnoses are known to be associated with MS [14, 15].

### Study outcomes and statistical analyses

The outcomes of the study were the initial diagnoses of MS within 10 years following the index date as function of IBD. Differences in the sample characteristics and diagnosis prevalence between IBD and non-IBD cohorts were compared using the Wilcoxon signed-rank test for continuous variables, the McNemar test for categorical variables with two categories, and the Stuart-Maxwell test for categorical variables with more than two categories. The 10-year cumulative incidence of MS in the cohort with and without IBD was further studied with Kaplan–Meier curves, and these curves were compared using the log-rank test. Finally, a univariable Cox regression analysis was conducted to assess the association between IBD and MS, whereas CD and UC were analyzed separately. Results of the Cox regression model are displayed as hazard ratios (HRs) and 95% confidence intervals (CIs). Additionally, Cox regression analyses were conducted separately for men, and women. A *p*-value of < 0.05 was considered to be statistically significant. Analyses were carried out using SAS version 9.4 (SAS Institute, Cary, USA).

## Results

### Basic characteristics of the study sample

The present study included 24,934 individuals with IBD (11,140 with CD and 13,794 with UC) and 24,934 individuals without IBD. The basic characteristics of study patients are displayed in Table 1. Mean age was 50.3 years (standard deviation (SD): 18.5 years). 55.6% of patients were female. The average annual frequency of GP visits was 9.4 times during the follow-up. Due to the matched pairs design, no significant differences were observable between both cohorts in terms of age, sex, visit frequency, and predefined co-diagnoses including diabetes, obesity, vitamin D deficiency, and autoimmune disorders (Table 1).

**Table 1:** Baseline characteristics of the study sample (after propensity score matching)

Variable	Proportion among Individuals with IBD (%) *N* = 24,934	Proportion among individuals without IBD (%) *N* = 24,934	*p*-value
Age (Mean, SD)	50.3 (18.5)	50.3 (18.5)	0.921
Age 18–30	19.3	19,1	0.967
Age 31–40	14.2	14.3	
Age 41–50	17.4	17.3	
Age 51–60	17.9	17.9	
Age > 60	31.2	31.4	
Women	55.6	55.6	0.907
Men	44.4	44.4	
Number of physician visits per year during the follow-up (Mean, SD)	9.4 (8.6)	9.3 (8.7)	0.086
Diabetes	18.2	18.3	0.826
Obesity	9.1	8.9	0.348
Vitamin D deficiency	3.4	3.4	0.980
Autoimmune disorders^a^	15.5	15.5	0.921

Proportions of patients given in %, unless otherwise indicated

*SD* standard deviation

^a^Autoimmune disorders include rheumatoid arthritis, psoriasis, atopic dermatitis, autoimmune thyroiditis, ankylosing spondylitis, systemic lupus erythematosus, celiac disease, thrombophilia

### Crohn’s disease and ulcerative colitis are associated with an increased incidence of multiple sclerosis

**Fig. 2:**
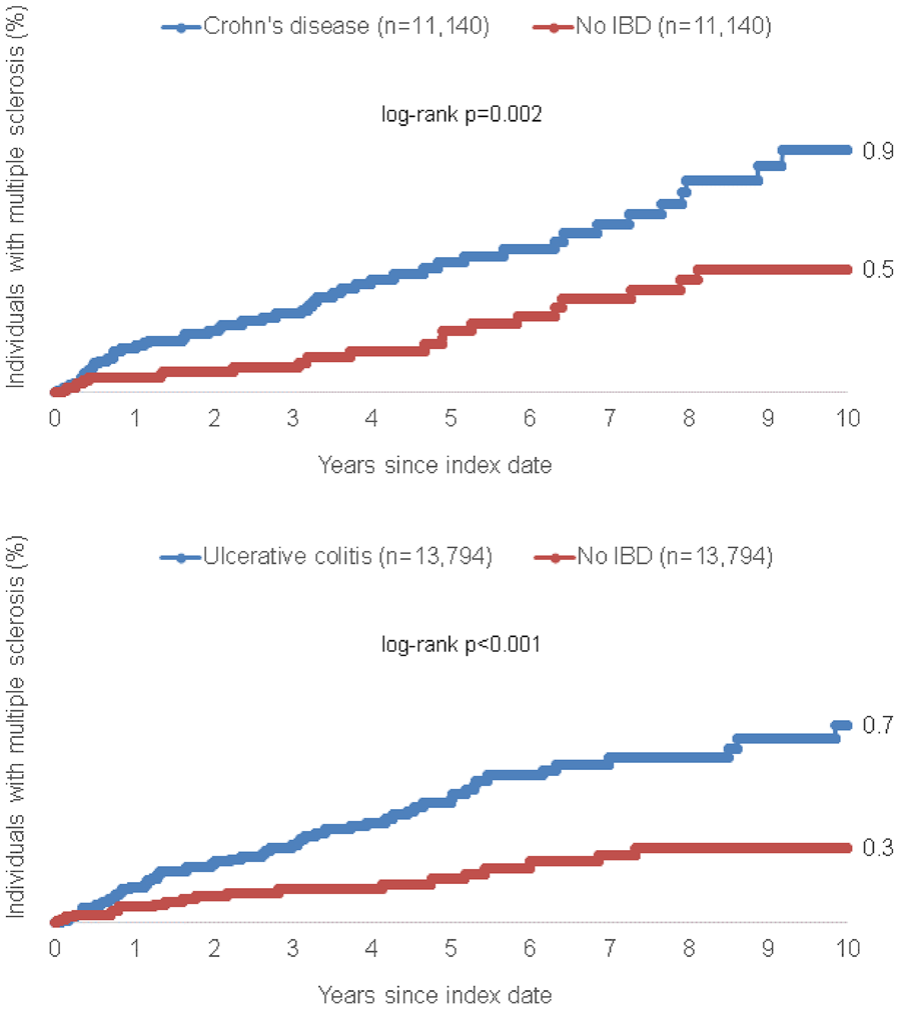
Cumulative incidence of multiple sclerosis in individuals with and without Crohn’s disease and ulcerative colitis

After 10 years of follow-up, 0.9% of CD patients but only 0.5% of matched non-IBD pairs were diagnosed with MS (*p* = 0.002, Fig. 2). In addition, the incidence of MS was significantly higher among UC patients (0.7%) compared to matched non-IBD patients (0.3%) within the 10 years observation period (*p* < 0.001, Fig. 2). Univariate Cox-regression analysis confirmed a significant association between both CD (HR: 2.09; 95% CI 1.28–3.39) as well as UC (HR: 2.35; 95% CI 1.47–3.78) and MS (Table 2).

**Table 2:** Association between Crohn’s disease and ulcerative colitis and subsequent Multiple Sclerosis in patients followed in general practices in Germany (univariable Cox regression models)

Patient group	Crohn’s disease		Ulcerative colitis	
	HR (95% CI)	*p*-value	HR (95% CI)	*p*-value
Total	2.09 (1.28–3.39)	0.003	2.35 (1.47–3.78)	< 0.001
Women	1.72 (0.99–2.98)	0.055	1.95 (1.11–3.43)	0.012
Men	3.92 (1.32–11.66)	0.014	3.60 (1.46–8.85)	0.005

### The association between IBD and MS is more pronounced among males

In a next step, we compared the subsequent incidence of MS among IBD patients for male and female patients separately. Cox-regression analyses revealed that the association between CD and MS was only significant among male patients (HR: 3.92; 95% CI 1.32–11.66, *p* = 0.014) but not female patients (HR: 1.72; 95% CI 0.99–2.08, *p* = 0.055, Table 2). Similarly, the association between UC and MS was more pronounced among male patients (HR: 3.60; 95% CI 1.48–8.85) compared to female patient (HR: 1.95; 95% CI 1.11–3.43, Table 2).

## Discussion

The present study provides further evidence for an epidemiological association between IBD and a subsequent diagnosis of MS in a cohort of primary care patients in Germany. In addition, this association was stronger in men than in women. Our data are in line with previous reports, showing an increased incidence of MS among IBD patients [16, 17]. Among these, a recently meta-analysis revealed a risk ratio of 1.91, which is comparable with our findings. Interestingly, the authors also observed an increased incidence of IBD among patients with MS, underlining the close pathophysiological link between the two conditions [16].

Reports exploring a possible relationship between MS and IBD date back to the early 1980s, whenan increased prevalence of MS was described for women who underwent total colectomy for UC or CD in the United Kingdom [18]. Subsequent studies where able to corroborate this association. A recent systematic review and meta-analysis highlighted not only an increased prevalence of MS in patients with IBD, but also a heightened prevalence of IBD in patients with MS [4]. Despite longstanding evidence for an epidemiological association between MS and IBD, the mechanisms behind this association are far from understood. This poses a significant challenge in disease management of patients with comorbid MS and IBD, as treatment options for one condition may adversely affect the other. Specifically, the use of interferon-β, a cytokine commonly used to treat MS, has been associated with increased severity of IBD [19]. Of note, the evidence regarding the effect of interferon treatment on symptoms in certain patients with IBD is equivocal, with some studies suggesting a potential benefit [19, 20]. In contrast, TNF-α antagonists, which are effective for treating IBD, have been shown to worsen the severity of MS [9, 21]. While treatments with TNF-α antagonists were debated as one possible explanation for the observed epidemiological association between IBD and MS, prior studies reported an increased risk for subsequent MS diagnosis even in the absence of anti-TNF-α therapy. For instance, one study analyzing white matter hyperintensities was able to demonstrate a significantly higher incidence of white matter T2-hyperintensities in patients with CD compared to controls, despite displaying no correlation to anti-TNF therapy [22]. Furthermore, in a retrospective analysis examining a total of 9095 IBD patients, anti-TNF-α therapy did not increase the risk of subsequent diagnosis of clinical idiopathic inflammatory demyelinating disease [23]. Finally, it is noteworthy that natalizumab, a highly specific alpha4-integrin antagonist approved for the treatment of MS [24], has also been shown to induce clinical remission in patients with moderately to severely active CD [25, 26], suggesting that synergistic effects may be useful in patients with CD and MS. However, natalizumab is not approved for the treatment of CD in Europe [27].

In recent years, shared genetic patterns in autoimmune diseases—and in particular between MS and IBD—have gained rising attention as a possible contributor to the observed association between these two conditions. Besides an increased familial occurrence of autoimmune disorders in general and overlapping genetic loci [28], there is also a growing number of identified shared genetic polymorphisms between MS and IBD [6, 29]. For instance, one study was able to identify common genetic polymorphisms in CD4 + T cells and CD8 + cytotoxic T cells that may contribute to the comorbid association between MS and IBD [30]. Specifically, IL23A, a gene involved in CD4 + T cell function, has been implicated in the pathophysiology of both MS and IBD. In addition, PTGER4—encoding the prostaglandin E_2_ receptor 4—has been found to be involved in susceptibility to both MS and CD and was highly expressed in CD8 + cytotoxic T cells. These findings suggest a possible role for these cell types and genes in shared immune regulation pathways that contribute to the development of MS and IBD.

Besides genetic predisposition, gut microbial dysbiosis and dysfunction of the intestinal barrier have been linked to the onset and progression of MS [31, 32]. Of note, in context of both MS and IBD, a significant association with the prevalence of Epstein-Barr virus (EBV) has been reported. In MS, the risk of disease initiation was significantly increased after infection with EBV, but not other similarly transmitted viruses such as cytomegalovirus [33]. For IBD, we were able to show a similar significant association with infectious mononucleosis and CD in a previous study [34]. Interestingly, this relationship was only observed in female patients. In contrast, the present study demonstrates a significant association between CD and MS only in male patients. While sex-differences in comorbidities of MS have been described before, these studies have established higher prevalence of IBD in female patients with MS, although statistical significance was not reached [8]. In general, the role of sex hormones is of critical importance in autoimmune and inflammatory diseases such as IBD or MS. For instance, testosterone has been shown to exert protective effects and to improve inflammatory markers regarding various autoimmune diseases [35]. In the case of MS, higher serum levels of testosterone may decrease pro-inflammatory cytokines, such as TNF-α and INF-γ, and induce a shift in T helper cell (Th) profile from a Th1-dominant to an Th2-dominant type, while also suppressing lymphocyte proliferation and immunoglobulin production [36].

One other potential commonality between MS and IBD is vitamin D deficiency. Vitamin D which is an essential nutrient regulating the immune system and reducing inflammation has been shown to improve flares in IBD and disease progression in MS [37, 38]. Furthermore, sufficient serum vitamin D levels have been shown to decrease the risk of developing MS or IBD [39, 40]. The connection between Vitamin D and MS and IBD, however, is still not very well understood even though sufficient intake of vitamin D may improve both conditions.

There are several limitations to our study that must be considered. First, in an outpatient treatment setting, we acknowledge that some diagnoses may be subject to incorrect coding or misclassification by the attending physician. Second, the Disease Analyzer database does not provide data on pertinent factors such as the socioeconomic status of patients (e.g., education and income) or lifestyle-related risk factors (e.g., smoking, alcohol consumption and physical activity) which may potentially introduce bias to our analysis. Third, there was no available information regarding the methods of diagnosis of either MS or IBD, and it is possible that diagnoses recorded by the general practitioner in charge may have been previously made by a specialist or in a hospital setting without the GP's involvement in the diagnostic process. Fourth, in GP practices, no information on the individual therapy for IBD such as biological prescription was recorded as this therapy is usually given by gastroenterologists. Therefore, our study cannot answer the important question of whether or not, e.g., anti-TNF treatment might influence the incidence of MS in our cohort of patients. Fifth, the dataset does not contain detailed information on the presence of other extraintestinal disease manifestation of IBD or the particular phenotypes of IBD and MS. Sixth, the matching design used in the study, can cause the so-called overmatching what can lead to an increased similarity between cases and controls in terms of exposure levels, with a consequence what a study population can become not representative of the broader population. Lastly, due to the study design, we can only make assumptions about associations between variables and cannot infer any causal relationships.

In summary, this study provides evidence for a significant association between IBD and subsequent diagnosis of MS in a large outpatient cohort in Germany. These findings may provide important implications for the management and treatment of patients with IBD and MS, although future studies are needed to, e.g., evaluate the specific impact of IBD treatment on this association.

## Data Availability

The underlying data are available upon reasonable request from the corresponding author.
